# Conformational Change of Amyloid-*β* 40 in Association with Binding to GM1-Glycan Cluster

**DOI:** 10.1038/s41598-019-43117-6

**Published:** 2019-05-02

**Authors:** Yuhei Tachi, Yuko Okamoto, Hisashi Okumura

**Affiliations:** 1 Nagoya University, Department of Physics, Graduate school of Science, Nagoya, Aichi 464-8602 Japan; 2 National Institutes of Natural Sciences, Research Center for Computational Science, Institute for Molecular Science, Okazaki, Aichi 444-8585 Japan; 3 Nagoya University, Structural Biology Research Center, Graduate School of Science, Nagoya, Aichi 464-8602 Japan; 4 Nagoya University, Center for Computational Science, Graduate School of Engineering, Nagoya, Aichi 464-8603 Japan; 5 Nagoya University, Information Technology Center, Nagoya, Aichi 464-8601 Japan; 6 JST-CREST, Nagoya, Aichi 464-8602 Japan; 7 SOKENDAI (The Graduate University for Advanced Studies), Department of Structural Molecular Science, Okazaki, Aichi 444-8585 Japan; 8 National Institutes of Natural Sciences, Exploratory Research Center on Life and Living Systems, Okazaki, Aichi 444-8585 Japan

**Keywords:** Biological physics, Computational biophysics

## Abstract

Aggregates of amyloid-*β* (A*β*) peptide are well known to be the causative substance of Alzheimer’s disease (AD). Recent studies showed that monosialotetrahexosylganglioside (GM1) clusters induce the pathological aggregation of A*β* peptide responsible for the onset and development of AD. However, the effect of GM1-glycan cluster on A*β* conformations has yet to be clarified. Interactions between A*β* peptide and GM1-glycan cluster is important for the earliest stage of the toxic aggregation on GM1 cluster. Here, we performed all-atom molecular dynamics (MD) simulations of A*β*40 on a recently developed artificial GM1-glycan cluster. The artificial GM1-glycan cluster facilitates the characterization of interactions between A*β*40 and multiple GM1-glycans. We succeeded in observing the binding of A*β*40 to the GM1-glycan cluster in all of our MD simulations. Results obtained from these MD simulations indicate the importance of HHQ (13-15) segment of A*β*40 for the GM1-glycan cluster recognition. This result is consistent with previous experimental studies regarding the glycan recognition of A*β* peptide. The recognition mechanism of HHQ (13-15) segment is mainly explained by non-specific stacking interactions between side-chains of histidine and rings of sugar residues, in which the HHQ regime forms coil and bend structures. Moreover, we found that A*β*40 exhibits helix structures at C-terminal side on the GM1-glycan cluster. The helix formation is the initial stage of the pathological aggregation at ceramide moieties of GM1 cluster. The binding of Lys28 to Neu triggers the helix formation at C-terminus side because the formation of a salt bridge between Lys28 and Neu leads to change of intrachain interactions of A*β*40. Our findings suggest that the pathological helix formation of A*β*40 is initiated at GM1-glycan moieties rather than lipid ceramide moieties.

## Introduction

Amyloid-*β* (A*β*) peptide, which is one of intrinsically disordered proteins (IDPs) in aqueous solution, is believed that its misfolded aggregates, namely A*β* oligomers and fibrils, cause Alzheimer’s disease (AD) in human brain^1–5^. Recent experimental studies indicate that monosialotetrahexosylganglioside (GM1) in neuronal cell membranes is involved in the toxic aggregation of A*β* peptides^6–10^. GM1 is an abundant glycosphingolipids in neuronal cell membranes, and GM1 clustering promotes physiological and pathological functions including the onset and development of AD^6–8,11,12^. In fact, GM1 bound to A*β* (GA*β*) was experimentally found in AD human brain by Hayashi *et al*.^13^. Previous works reported that GM1 clusters specifically interact with A*β* peptides and trigger folding and aggregation which leads to the toxic fibrils and oligomers^14–16^. Hence, understanding the interactions between A*β* peptide and GM1 cluster is essential in the therapeutic context of AD^17^.

To clarify the interaction between A*β* peptide and GM1 cluster, experimental studies revealed structural characteristics of A*β* peptide on GM1 cluster using artificial GM1 enviroments (e.g., micells and liposomes)^18–22^. These studies showed that A*β* peptides form helical structures at the hydrophobic environment provided by lipid hydrocarbon chains of GM1 micells^18,19,22^. A further study indicated that A*β*-A*β* interactions coupled with the formation of Thioflavin T-reactive *β*-structures are promoted where the A*β* density is high in GM1 micells^20^. A computational study of A*β*42 and GM1 containing lipid bilayer supports the exhibition of *β*-structures^23^. Besides these experimental studies^18–20^, K. Ikeda *et al*. showed that *α*-to-*β* transition is caused by changing the ratio of A*β* over GM1 using liposomes^21^. In this transition, only helical structures exist at lower A*β* densities, and aggregated *β*-structures increase more than monomeric helical structures at higher A*β* densities. A series of studies^18–22^ indicate that *α*-to-*β* transition on GM1 cluster is a part of the pathological aggregation pathways, and emphasize the importance of lipid hydrocarbon chains. However, the effect of GM1-glycan cluster on A*β* conformations is poorly understood. Interactions between GM1-glycan cluster and A*β* peptide play an important role in the earliest stage of the A*β* aggregation process on GM1 clusters.

Recently, an artificial GM1-glycan cluster based on a metal-ligand complex has been proposed to characterize the GM1-glycan recognition of A*β*40 by nuclear magnetic resonance (NMR) analyses^24^. The metal-ligand complex consists of 24 bent ligands and 12 palladium ions, and provides a stable scaffold for well-defined GM1-glycan cluster^25,26^. The GM1-glycan cluster lacking in lipid ceramide moieties is a useful tool to characterize the effects of GM1-glycan moieties on amyloidgenic proteins. This previous study suggested that A*β*40 recognizes the GM1-glycan cluster using its N-terminal side^24^. The N-terminal side recognizes various types of glycan environments, such as GM1 micells^27,28^, heparin^29,30^, and microglia cells^31^. However, the N-terminal side selectivity of these glycan environments can not be explained by simple glycan multivalent interactions with A*β* peptides. It motivates us to investigate interactions between the GM1-glycan cluster and A*β* peptide at atomic level. Especially, an important question regarding the pathological context of AD is which conformations of A*β* peptide are adopted to recognize the GM1-glycan cluster. The conformational information of A*β* peptide bound to GM1-glycan cluster is poteintially important for logical designing of drug materials and self-assembled supramolecules.

Complementarily to experiments, molecular dynamics (MD) simulations have been utilized to characterize molecular systems related to AD^22,23,32–37^. In our previous study, to elucidate the interactions between A*β* peptide and the GM1-glycan cluster, we first modeled the GM1-glycan cluster and investigated its structural characteristics using all-atom MD simulations^38^. Here, we report results of further MD simulations of A*β*40 on the GM1-glycan cluster. In addition, we performed MD simulations of monomeric A*β*40 to compare the conformational ensemble of monomeric A*β*40 and that of A*β*40 on the GM1-glycan cluster. This comparison helped us to characterize the conformational ensemble of A*β*40 on the GM1-glycan cluster. In this study, MD simulations demonstrate the adhesion of A*β*40 to the GM1-glycan cluster. Our results explain the reason of the N-terminal side selectivity for the GM1-glycan cluster recognition at atomic level. Furthermore, we found that the helix formation of A*β*40 in association with the GM1-glycan cluster recognition, and proposed the mechanism of this conformational change. These results provide physicochemical insights for understanding the earliest stage of the pathological aggregation process on GM1 cluster.

## Results and Discussion

**Figure 1:**
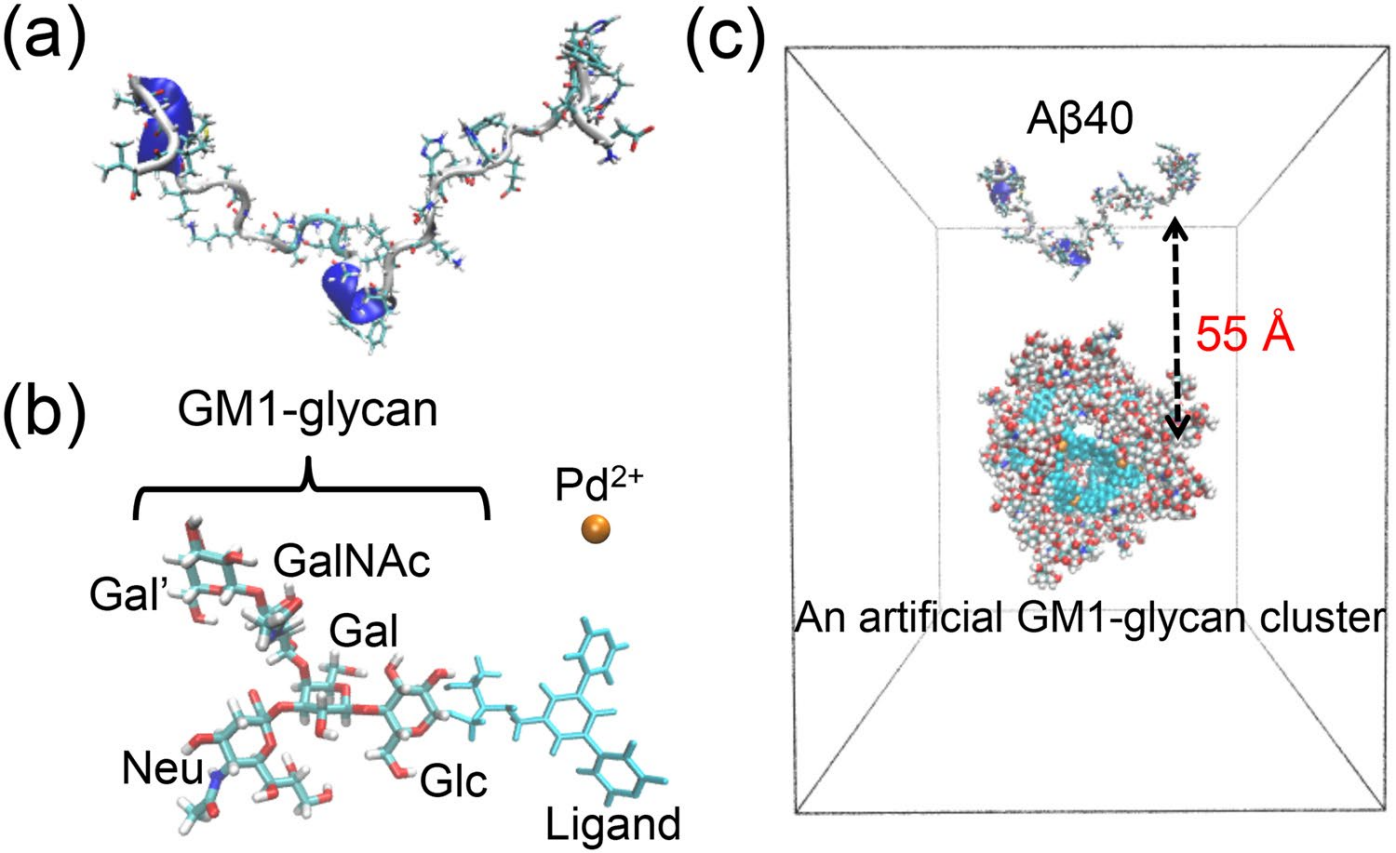
(**a**) An initial conformation of A*β*40 for binding to the GM1-glycan. (**b**) Structures of ligand with GM1-glycan and palladium ion. Ligand was introduced to connect to Glc of GM1-glycan instead of the lipid ceramide moiety^24^. (**c**) An initial position of A*β*40 and an artificial GM1-glycan cluster.

All-atom MD simulations of A*β*40 on the GM1-glycan cluster and monomeric A*β*40 were performed with nine different initial structures and velocities for 1.5 *μ*s. An initial conformation of A*β* peptide for binding to the GM1-glycan cluster was extracted from the last trajectory of a 100 ns MD simulation (see Fig. 1a). The GM1-glycan cluster is composed of 24 ligands with GM1-glycan and 12 palladium ions (see Fig. 1b). An initial conformation for the GM1-glycan cluster was extracted from the last trajectory of a 250 ns MD simulation of our previous study^38^. A*β* peptide was placed at a position of 55 Å from the center of mass of the GM1-glycan cluster in each initial structure (Fig. 1c). A*β*40 on the GM1-glycan cluster and monomeric A*β*40 were solvated with water molecules. The number of water molecules were 31083–31103 and 12998–13020 for A*β*40 on the GM1-glycan cluster and monomeric A*β*40, respectively. Temperature was set to 300 K, which is the experimental condition^24^.

### Formation of GM1-glycan cluster bound to A*β*40

**Figure 2:**
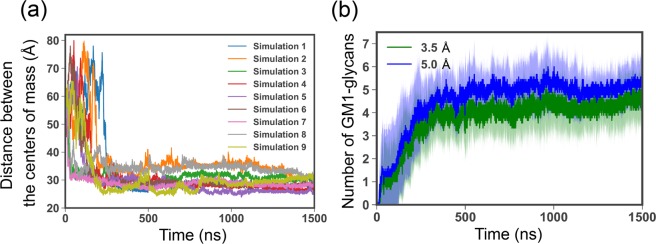
(**a**) Time series of distance between centers of mass of A*β*40 and the GM1-glycan cluster in each different trajectory. (**b**) Time series of average number of GM1-glycans bound to A*β*40 with different cutoff distances, 3.5 and 5.0 Å. Dark color and light color mean average value and sample standard deviation at each time, respectively.

First, the formation of GM1-glycan cluster bound to A*β*40 must be confirmed in this study. We therefore calculated time series of the distance between centers of mass of A*β* peptide and the GM1-glycan cluster in each different trajectory (see Fig. 2a). To calculate the centers of mass, non-hydrogen atoms were considered. This figure shows that A*β*40 binds to the GM1-glycan cluster at ~30 Å after ~300 ns in each MD simulation. Figure 2b shows time series of average number of GM1-glycans bound to A*β* peptide. These were calculated by using different cutoff distances, 3.5 and 5.0 Å (Fig. 2b). We regarded GM1-glycan as being bound to A*β* when a minimum distance between non-hydrogen atoms of the GM1-glycan and those of A*β* was less than the cutoff distance. Figure 2b shows that multiple GM1-glycans gradually bind to A*β* peptide and the average numbers converge in both cutoff distances after ~500 ns MD simulations. This result indicates that multiple GM1-glycans are used to capture A*β* peptide. It corresponds to glycan-protein multivalent interactions. Thus, we considered to be equilibrated the conformation of GM1-glycan cluster bound to A*β* after ~500 ns simulation. Hereafter, we calculated all quantities from the last 750 ns of each MD simulation.

### Importance of HHQ (13-15) regime for the GM1-glycan cluster recognition

**Figure 3:**
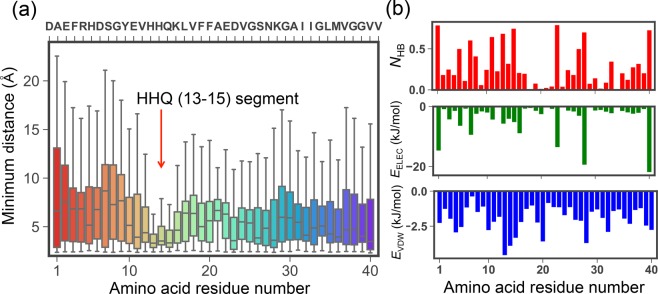
(**a**) Boxplots of minimum distance between each amino acid residue of A*β*40 and the GM1-glycan cluster. (**b**) The average values of the number of hydrogen bonds *N*_HB_ (upper figure), electrostatic interaction energy *E*_ELEC_ (middle figure), and van der Waals (VDW) interaction energy *E*_VDW_ (lower figure) between each amino acid residue and the GM1-glycan cluster.

**Figure 4:**
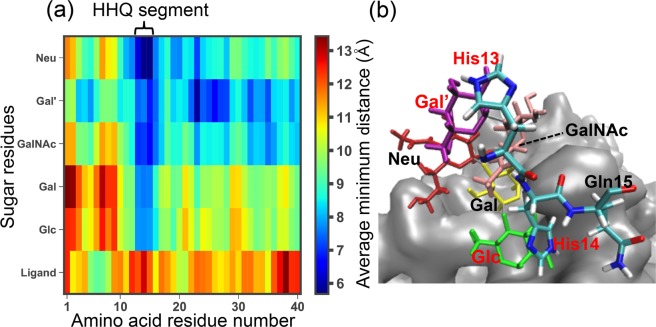
(**a**) Matrix of average minimum distance between each amino acid residue and each sugar residue including Ligand. (**b**) A typical snapshot of the HHQ segment and GM1-glycan. Silver colored molecule contains the other GM1-glycans, Ligands, and palladium ions.

To analyze the glycan cluster recognition site of A*β*40, we calculated boxplots of minimum distance between each amino acid residue and the GM1-glycan cluster (see Fig. 3a). Non-hydrogen atoms were used on the calculation of minimum distances. This figure shows that the HHQ (13–15) regime closely interacts with the GM1-glycan cluster compared to the other amino acids. It is consistent with the experimental result that A*β*40 recognizes the GM1-glycan cluster by its N-terminal side^24^. Previous computational study also reported the importance of the HH (13-14) regime^39^. Interestingly, previous experimental works indicated that the HHQ regime recognizes the other glycan environments, e.g., GM1 micells^27,28^, heparin^29,30^, and microglia cells^31^. These results imply that A*β* peptide recognizes the glycan environments in a similar manner. To investigate interactions between the HHQ regime and GM1-glycan in more detail, we calculated the average values of the number of hydrogen bonds, electrostatic interaction energy, and van der Waals (VDW) interaction energy between each amino acid residue and the GM1-glycan cluster (see Fig. 3b). A hydrogen bond was regarded as being formed when *X*_AD_ < 3.5 Å and ***θ***_AHD_ > 135° (*X*_AD_ is the distance between accepter (A) and donor (D) heavy atoms and ***θ***_AHD_ is A-H-D angle) was satisfied. This figure indicates that the electrostatic interaction energies of charged residues contributes largely with hydrogen bonds; however, VDW interaction energies correlates with the approach of the HHQ regime more than electrostatic interaction energies. The matrix of average minimum distance between each amino acid residue and each sugar residue including Ligand is shown in Fig. 4a. A value of average minimum distance between an amino acid residue and a sugar residue was defined by ${\sum }_{i=1}^{N}\,{x}_{i}/N$ where *x*_*i*_ is the minimum distance at the *i*-th frame of the  subsampled MD trajectories, and *N* is the frame number of the subsampled MD trajectories. In this figure, each amino acid residue except the HHQ regime tends to be closer to the outer layer sugar residues, Neu, Gal’, and GalNAc. On the other hand, the HHQ regime is close to the inner layer sugar residues (Gal and Glc) as well as the outer layer sugar residues. Why only the HHQ regime can be non-specifically close to sugar residues? A typical snapshot of the HHQ regime and GM1-glycan was shown in Fig. 4b. In this snapshot, it can be seen that imidazole group of His13 and His14 are stacked with rings of Gal and Glc. Actually, several studies have reported that the ring of galactose interacts with hydrophobic or aromatic side-chains of amino acids^40–42^. Thus, we concluded that the HHQ (13–15) regime mainly recognizes the GM1-glycan cluster by stacking with rings of sugar residues. Its stacking interactions allow that the HHQ regime non-specifically interacts with sugar residues. Moreover, this non-specific recognition mechanism of HHQ regime against sugar residues can explain the reason why the HHQ regime widely recognizes glycan environments, such as GM1 micells^27,28^, heparin^29,30^, and microglia cells^31^.

### Major conformations of GM1-glycans bound to the HHQ segment

We investigated major conformations of GM1-glycans bound to the HHQ segment using the dihedral angle principal component analysis (dPCA). Figure S1 of Supplementary Information (SI) shows scatter plots as a function of the first two eigenvectors PC1 and PC2 and the second and third eigenvectors PC2 and PC3 in the cases of only GM1-gycan and the HHQ segment bound to the GM1-glycan. Each eigenvector was calculated from the data set of our previous MD simulations^38^. We considered that GM1-glycan bound to the HHQ regime when minimum distance between the GM1-glycan and the HHQ regime is less than 5.0 Å since Fig. 3a shows that most samples of GM1-glycans bound to the HHQ regime are within 5.0 Å. From this figure, although six major clusters are observed as in that of the previous MD simulations in the case of only GM1-glycan, clusters 5 and 6 have lower densities in the case of HHQ segment bound to GM1-glycan. Figure S2 of SI shows conformations of each cluster and the end-to-end distances. Clusters 5 and 6 have shorter end-to-end distances than those of the other clusters, due to the bending conformations. Therefore, we consider that the bending conformations of clusters 5 and 6 inhibit to interact with histidine residues by stacking interactions.

### Pathological Helix formation of A*β*40 on the GM1-glycan cluster

**Figure 5:**
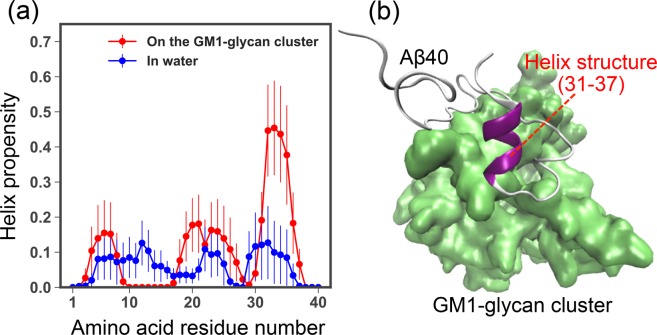
(**a**) Helix propensities in each amino acid residue assigned by the DSSP algorithm^44^. The Jackknife method^45^ was used for estimating the average values and standard errors from correlated time series. (**b**) A typical snapshot of A*β*40 with a helix structure formed in residues 31–37. Green colored molecule is the GM1-glycan cluster.

Helix formation is the initial stage of the pathological aggregation process on GM1 cluster. We then calculated the helix propensities in each amino acid residue using DSSP (Define Secondary Structure of Proteins) algorithm^43^ (see Fig. 5a). This calculation was carried out in the cases of A*β*40 on GM1-glycan cluster and monomeric one. We estimated the average values and standard errors from correlated time series by using Jackknife method^44^. This figure shows that the GM1-glycan cluster environment significantly stabilizes helix structures at residues 31–37 compared to that of monomeric one. Whereas, the helix formation of the HHQ regime is destabilized on the GM1-glycan cluster. A typical snapshot of A*β* peptide that formed a helix structure at residues 31–37 is shown in Fig. 5b. Figure S3 of SI shows the two dimensional free energy landscape *F*(*d*, *c*) as a function of helix content *c* and the distance *d* between centers of mass of A*β* peptide and the GM1-glycan cluster. Values of the helix content distributes up to ~30% at ~30 Å in this figure. A previous experimental CD study demonstrated that residues 31–36 of A*β* peptide form helical structures and the helix content is ~23% at hydrophilic/hydrophobic interfaces of GM1 micells^18^. A recent study combined NMR and MD simulations also showed the helix formation at residues 31–36 on GM1 micells^22^. Besides the helix formation, a previous computational study showed that A*β* peptide binds to GM1-glycan moieties rather than lipid ceramide moieties of GM1 cluster at first^23^. Therefore, these results suggest that the pathological helix formation of A*β*40 initially can be triggered by GM1-glycan moieties in the A*β* aggregation process.

### Binding Lys28 to Neu plays a key role in the pathological helix formation

**Figure 6:**
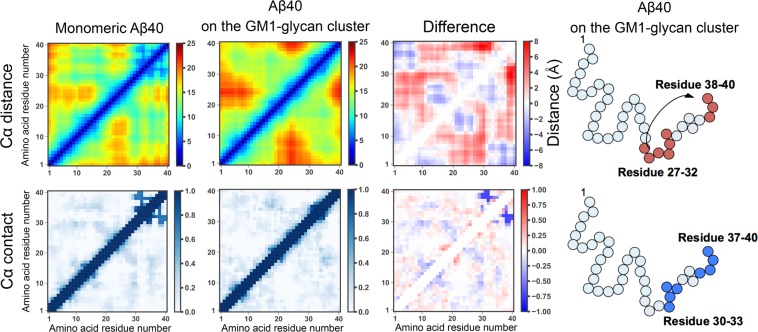
Matrices of average C*α* distance and contact in each residue pair in the cases of A*β*40 on the GM1-glycan cluster and monomeric A*β*40, and these matrix differences.

**Figure 7:**
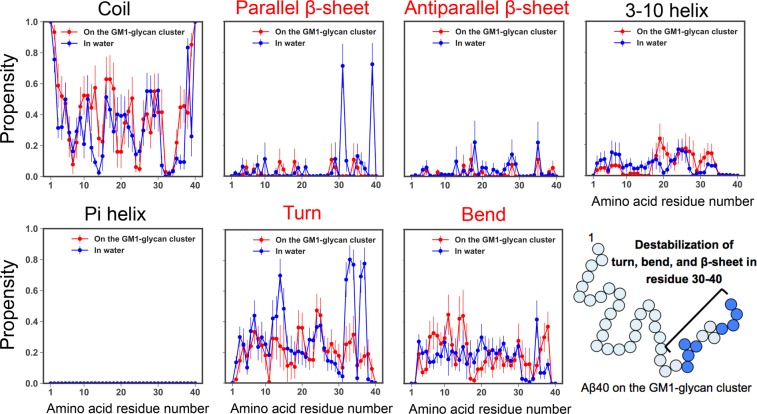
Propensities of each secondary structure except the helix structure in each amino acid residue assigned by the DSSP algorithm^44^. The average values and standard errors were estimated by the Jackknife method^45^.

**Figure 8:**
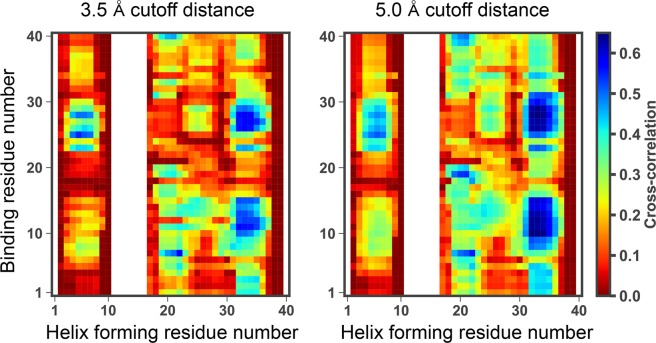
Matrices of cross-correlation *C*_*ij*_ between helix formation and binding to the GM1-glycan cluster in each amino acid residue with different cutoff distances, 3.5 and 5.0 Å. These values were calculated from all of the subsampled MD trajectories. White color means that residues correspond to the abscissa did not form helix structures during all MD simulations.

**Figure 9:**
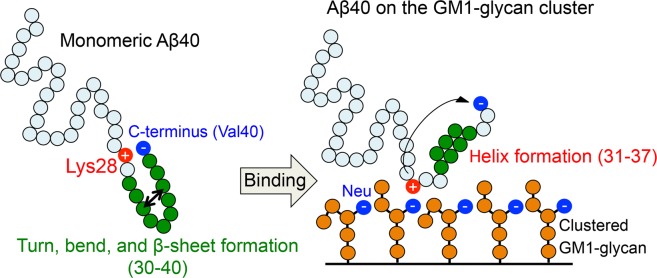
A schematic figure of the helix formation mechanism of A*β*40 at residues 31–37 on the GM1-glycan cluster.

The question that attracts our physicochemical interests is how these helix structures are notably stabilized on the GM1-glycan cluster. To understand this molecular mechanism, we calculated matrices of average C*α* distance and contact in each residue pair in the cases of A*β*40 on the GM1-glycan cluster and monomeric one in water, and matrix differences between the two cases (see Fig. 6). A C*α* contact was regarded as being formed when the distance between C*α* atoms was less than 8.0 Å. Each difference between the *i*-th residue and the *j*-th residue was defined by simple subtraction of average values, ${\rm{Difference}}\,(i,j)=\langle {x}_{ij}^{\mathrm{with}-\mathrm{glycan}}\rangle -\langle {x}_{ij}^{\mathrm{only}-A\beta }\rangle$, where *x* corresponds to C*α* distance (in Å) or contact (1 or 0), $\langle {x}_{ij}^{\mathrm{with}-\mathrm{glycan}}\rangle$ and $\langle {x}_{ij}^{\mathrm{only}-A\beta }\rangle$ denote the average values between the *i*-th residue and the *j*-th residue in the cases of A*β*40 on GM1-glycan cluster and monomeric one, respectively. This figure shows that the C*α* distance between residues 38–40 and residues 27–32 increases, and simultaneously the number of C*α* contacts between residues 37–40 and residues 30–33 decreases on the GM1-glycan cluster. Propensities of each secondary structure except the helix structure are shown in Fig. 7. On the GM1 glycan cluster, propensities of turn, bend, and *β*-sheet structures mainly decrease instead of stabilizing helix structures in the C-terminal side. The destabilization of these secondary structures is involved with the change of the C*α* distance, because the increasing of the C*α* distance between residues 38–40 and residues 27–33 leads to decrease the number of C*α* contacts between residues 37–40 and residues 30–33. In regard to the HHQ regime, coil and bend structures are stabilized on the GM1-glycan cluster. It implies that the GM1-glycan cluster is a preferable environment for the HHQ regime than water solvents. Furthermore, we calculated matrices of cross-correlation *C*_*ij*_ between helix formation and binding to the GM1-glycan cluster in each amino acid residue using different cutoff distances, 3.5 and 5.0 Å (see Fig. 8). All frames of subsampled MD trajectories are used for the cross-correlation calculations. This figure shows that not only the HHQ regime, but also the binding of residues 26–29 strongly correlate with the helix formation on the GM1-glycan cluster. Here, note that positively charged Lys28 forms salt bridges with negatively charged Neu and C-terminus. Hence, we inferred that binding Lys28 to Neu of GM1-glycans triggers to increase the C*α* distance between residues 37–40 and residues 24–30 by inhibiting a salt bridge between Lys28 and C-terminus. Actually, side-chain distance between Lys28 and C-terminus increases on the GM1 glycan cluster compared to that of monomeric A*β*40 (see Fig. S4 of SI). A schematic figure of this helix formation mechanism is shown in Fig. 9. Moreover, a typical MD trajectory, in which A*β*40 forms a helix structure at the C-terminal side, is shown in Movie 1. In fact, Supplementary Movie 1 of SI indicates that the approaching of Lys28 (red colored residue) to Neu (green colored residue) leads to the keeping of C-terminus (blue colored residue) away, and A*β* forms a helix structure at C-terminal side. This movie supports our hypothesis of the helix formation mechanism.

From the above results, we propose the helix formation mechanism as follows in two steps: (1) salt bridge formation between Lys28 and Neu by binding A*β*40 to GM1-glycans inhibits to form a salt bridge between Lys28 and C-terminus and (2) the helix structures are stabilized instead of turn, bend, and *β*-sheet structures because of increasing the C*α* distance between residues 38–40 and residues 27–32. In fact, a previous MD study showed that helix structures have lower energy compared to random coil structures on a simple polypeptide in water solvent^45^. The helix formation mechanism supports the initiation of the pathological folding by binding GM1-glycan moieties of GM1 cluster. It will be helpful information to understand the pathological aggregation of intrinsically disordered A*β* peptide.

## Conclusions

In this study, to clarify the interactions between A*β* peptide and GM1-glycan cluster, we performed all-atom MD simulations of A*β*40 on a recently developed artificial GM1-glycan cluster^24^. GM1-glycan cluster without the lipid hydrocarbon chains facilitates the characterization of interactions between A*β*40 and multiple GM1-glycans. Additionally, We carried out all-atom MD simulations of monomeric A*β*40 in water to compare conformations of A*β*40 on the GM1-glycan cluster and those of monomeric one.

We successfully observed the adhesion of A*β*40 to the GM1-glycan cluster in all of the MD simulations. From analyses of these MD trajectories, we revealed that A*β*40 mainly recognizes four major conformations of GM1-glycans by its HHQ (13-15) segment. This result is consistent with the previous NMR study of the GM1-glycan cluster^24^. The GM1-glycan recognition mechanism of the HHQ segment is explained by non-specific stacking interactions between side chains of histidine residues and rings of sugar residues, in which the HHQ regime forms coil and bend structures. It implies that the GM1-glycan cluster is preferable environment for the HHQ regime than water solvents. This interaction manner is different from that of simple glycan multivalent interactions with proteins. The non-specific interaction mechanism can apply to explain not only the GM1-glycan cluster recognition, but also the other glycan recognitions such as GM1 micells^27,28^, heparin^29,30^, and microglia cells^31^.

Furthermore, we found that A*β*40 exhibits helix structures in residues 31–37 and the helix content distributed up to ~30% on the GM1-glycan cluster. A previous experimental study indicated that A*β*40 forms helix structures in residues 31–36 and that the helix content is ~23% at the hydrophilic/hydrophobic environments of GM1 micells^18^. The helix formation is the initial stage of the pathological aggregation pathway. In addition, a previous computational study demonstrated that A*β* peptide first encounters GM1-glycan moieties of GM1 cluster. Thus, our results suggest the GM1-glycan moieties initially trigger the pathological helix formation rather than the lipid hydrocarbon chain moieties. The helix formation mechanism is proposed as two steps: (1) salt bridge formation between Lys28 and Neu by binding A*β* to GM1-glycans inhibits to form a salt bridge between Lys28 and C-terminus and (2) helix structures are stabilized instead of turn, bend, and *β*-sheet structures in C-terminal side because of increasing the C*α* distance between residues 37–40 and residues 24–30. This helix formation mechanism supports binding of A*β*40 to the GM1-glycan moieties initially induces the conformational change responsible for the toxic aggregation.

Our MD simulations provide the physicochemical information of interactions between A*β*40 and the GM1-glycan cluster. It will help to design drug materials for AD and self-assembled supramolecules at atomic level. Particularly, it may be one of potential strategies to develop an inhibitor to prevent the adhesion of the HHQ (13–15) segment of A*β*40 to GM1-glycan surfaces in human brain. The structural insights obtained from MD simulations contribute to understanding of the pathological glycan recognition and aggregation of A*β* peptide.

## Methods

### Molecular dynamics (MD) simulations

All-atom MD simulations of A*β*40 on the GM1-glycan cluster and monomeric A*β*40 were performed by the AMBER16 program package^46^. To avoid the initial condition dependency, MD simulations with nine different initial structures and velocities were carried out in both cases. We employed the GLYCAM 06 force field for the GM1-glycan part^47^, the general AMBER force field (GAFF) for the Ligand part^48^, the AMBER ff14SB for the A*β* peptide^49^, and the TIP3P model for the water molecules^50^. For the Pd^2+^-ligand coordination interaction, the 12–6 Lennard-Jones (LJ) non-bonded model was employed^51^, and a harmonic restraint with force constant of 10 kcal/(mol⋅Å^2^) was applied to the Pd^2+^-N distance as in our previous work^38^. Nitrate counterions (NO^3^) of the GM-glycan cluster system were neglected to simplify the computational model. To neutralize both systems, three hydronium ions were added. We first performed energy minimizations by using the steepest descent method and conjugate gradient method. After that, we performed heating MD simulations for 20 ps from 0 K to 300 K. Then, 2 ns MD simulations in *NPT* ensemble were performed at 1 atm. Pressure was controlled using the  Berendsen barostat with a pressure relaxation time of 1.0 ps. Temperature was controlled by using the Langevin thermostat with a collision frequency of 5.0 ps^−1^. Production MD simulations of A*β*40 on the GM1-glycan cluster and monomeric A*β*40 in *NVT* ensemble were performed for 1.5 *μ*s. Electrostatic interactions were treated by the particle-mesh Ewald (PME) method^52^. The non-bonded cutoff distance was set to 12 Å. To perform MD simulations with 2.0 fs timestep, the SHAKE algorithm was used for all bonds involving hydrogen atoms^53^. For analysis, the MD trajectory data were subsampled every 200 ps interval.

### Two dimensional free energy landscape

Two dimentinal free energy landscape as a function of helix content *c* and the distance *d* between centers of mass of A*β* peptide and the GM1-glycan cluster was defined by

1 $$F(d,c)=-\,{k}_{{\rm{B}}}T\,\mathrm{log}\,P(d,c)-{F}_{{\rm{\min }}}$$

where *k*_B_ is the Boltzmann constant, *T* is the simulation temperature, and *P*(*d*, *c*) is a probability distribution as a function of *d* and *c*. *F*_min_ is the minimum value of −*k*_B_*T* log*P*(*d*, *c*).

### Helix content

To directly compare helix contents obtained from circlar dichoism (CD) experiments to results from our MD simulations, we calculated the helix content *c* by using a calculated mean residue ellipticity [***θ***_calc_] at 222 nm. The unit of mean residue ellipticity is deg⋅cm^2^/dmol. It was derived by Khandogin and Brooks, and has been applied to model peptides of A*β*^37^. The helix content *c* was given by

2 $$c=\frac{[{{\boldsymbol{\theta }}}_{{\rm{calc}}}]-{[{\boldsymbol{\theta }}]}_{{\rm{coil}}}}{{[{\boldsymbol{\theta }}]}_{{\rm{hel}}}-{[{\boldsymbol{\theta }}]}_{{\rm{coil}}}}\times \mathrm{100,}$$

where [***θ***]_coil_ is the mean residue ellipticity of a complete random coil which corresponds to the value of 640, and [***θ***]_hel_ is the mean residue ellipticity of a complete helix structure: [***θ***]_hel_ = 42500(1 − 3/*N*), where *N* is the number of residues^54,55^. The calculated mean residue ellipticity [***θ***_calc_] at 222 nm, which depends on the total number and length of helix formed residues, is given by

3 $$[{{\boldsymbol{\theta }}}_{{\rm{calc}}}]=\frac{{[{\boldsymbol{\theta }}]}_{{\rm{hel}}}}{N}\sum _{i=1}^{{N}_{{\rm{hel}}}}({n}_{i}-k),$$

where *N*_hel_ is the number of helix formed fragment in the peptide, *n*_*i*_ is the number of helix formed residues in the *i*-th helix formed fragment, and *k* is a minimum number of helix formed residues required to produce the CD signal, which was set to the value of 3^54,56^. Here, the number of helix formed residues *n*_*i*_ was obtained by the DSSP algorithm^43^.

### Dihedral angle principal component analysis (dPCA)

We applied dPCA to GM1-glycans to characterize major conformations of GM1-glycan. This method was developed to describe rugged free energy landscapes of protein folding^57^. In the basic PCA, we can reduce the dimensionality of a high dimensional data set by using the covariance matrix as

4 $${\sigma }_{ij}=\langle ({q}_{i}-\langle {q}_{i}\rangle )({q}_{j}-\langle {q}_{j}\rangle )\rangle ,$$

where *q*_1_, …, *q*_*N*_ are values of the data set. Its *N* eigenvectors and eigenvalues are obtained by diagonalizing the covariant matrix. The eigenvectors and eigenvalues correspond to the collective modes of the data set and their amplitude, respectively. Hence, we can describe major collective modes by using some larger eigenvectors. The *i*-th principal component *μ*_*i*_ of the data set is given by the inner product:

5 $${\mu }_{i}={{{\boldsymbol{v}}}_{i}}^{T}({\bf{q}}-\langle {\bf{q}}\rangle )$$

where ***ν***_*i*_ is the eigenvector which corresponds to the *i*-th largest eigenvalue. In the dPCA method, we used the data set of sine/cosine-transformed dihedral angles to avoid the problem arising from the circularity of dihedral angle. In this study, we used dihedral angles of the glycosidic linkage instead of the peptide backbone as *q*_2*n*_ = sin*ϕ*_*n*_ and *q*_2*n*−1_ = cos*ϕ*_*n*_, where *ϕ* is dihedral angle of glycosidic linkage, *n* = 1, …, 2*N*, and *N* is the number of glycosidic linkages.

### Cross-correlation between helix forming and binding to the GM1-glycan cluster in each amino acid residue

To examine the correlation between helix formation and binding to the GM1-glycan cluster in each amino acid residue, we defined a cross-correlation function *C*_*ij*_ by

6 $${C}_{ij}=\frac{\langle {X}_{i}{Y}_{j}\rangle }{\sqrt{\langle {X}_{i}^{2}\rangle \langle {Y}_{j}^{2}\rangle }},$$

where *X*_*i*_ and *Y*_*j*_ are indicater functions for helix formation of the *i*-th residue and the binding of the *j*-th residue to the GM1-glycan cluster

7 $${X}_{i}=(\begin{array}{cc}1 & (\mathrm{Helix}\,\mathrm{formation})\\ 0 & (\mathrm{The}\,\mathrm{others})\end{array}$$

and

8 $${Y}_{j}=(\begin{array}{cc}1 & (\mathrm{Bound})\\ 0 & (\mathrm{No} \mbox{-} \mathrm{bound})\end{array}\mathrm{.}$$

Here, we regarded each amino acid residue as being bound to the GM1-glycan cluster when a minimum distance between non-hydrogen atoms of the GM1-glycan and those of each amino acid residue was less than cutoff distance. The range of the cross-correlation function *C*_*ij*_ is [0, 1].

## Supplementary information

LaTeX Supplementary File

Supplementary Information

Supplementary Movie 1
