# Supplementary material for: Conformational Change of Amyloid-β 40 in Association with Binding to GM1-Glycan Cluster
Source: Sci Rep. 2019 May 2;9:6853. doi: 10.1038/s41598-019-43117-6 (PMC6497634; doi:10.1038/s41598-019-43117-6)
Supplement: Supplementary file 2 — Supplementary Information [file 41598_2019_43117_MOESM2_ESM.pdf]

Supplementary information

Conformational Change of Amyloid-β 40 in Association with Binding to GM1-Glycan Cluster

Yuhei Tachi,[1,2] Yuko Okamoto,[1,3,4,5,6] Hisashi Okumura[2,7,8,*]

[1]*Department of Physics, Graduate school of Science, Nagoya University, Nagoya, Aichi 464-8602, Japan*

[2]*Research Center for Computational Science, Institute for Molecular Science, National Institutes of Natural Sciences, Okazaki, Aichi 444-8585, Japan*

[3]*Structural Biology Research Center, Graduate School of Science, Nagoya University, Nagoya, Aichi 464-8602, Japan*

[4]*Center for Computational Science, Graduate School of Engineering, Nagoya University, Nagoya, Aichi 464-8603, Japan*

[5]*Information Technology Center, Nagoya University, Nagoya, Aichi 464-8601, Japan*

[6]*JST-CREST, Nagoya, Aichi 464-8602, Japan*

[7]*Department of Structural Molecular Science, SOKENDAI (The Graduate University for Advanced Studies), Okazaki, Aichi 444-8585, Japan*

[8]*Exploratory Research Center on Life and Living Systems, National Institutes of Natural Sciences, Okazaki, Aichi 444-8585*

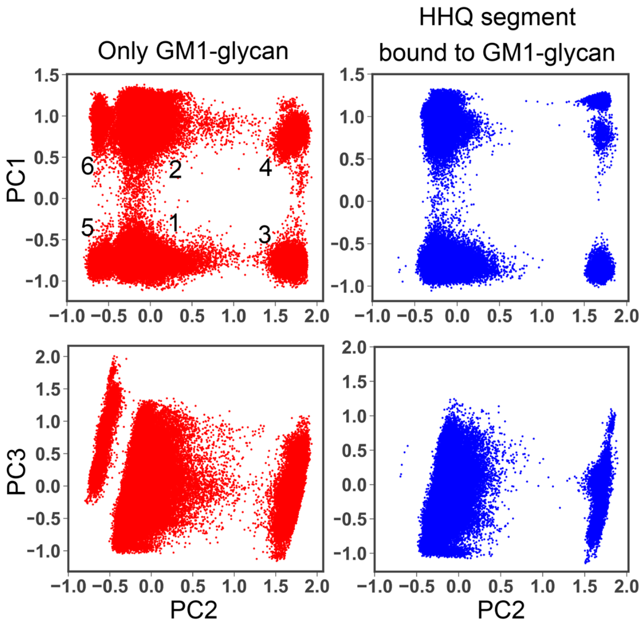

Figure S1. Scatter plots as a function of the first two eigenvectors PC1 and PC2 and the second and third eigenvectors PC2 and PC3 in the cases of only the GM1-glycan and the HHQ segment bound to the GM1-glycan.

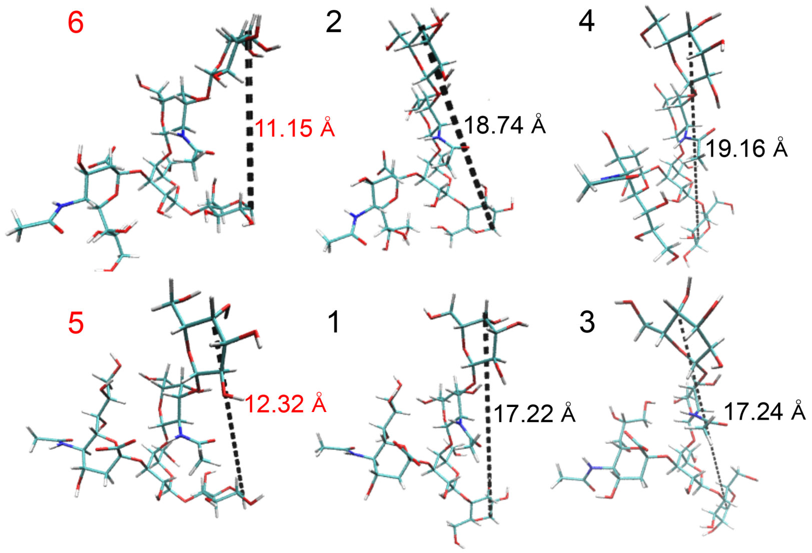

Figure S2. Conformations of each cluster and the end-to-end distances. The end-to-end distance was defined as a distance between C1 atom of Glc and C4 atom of Gal'.

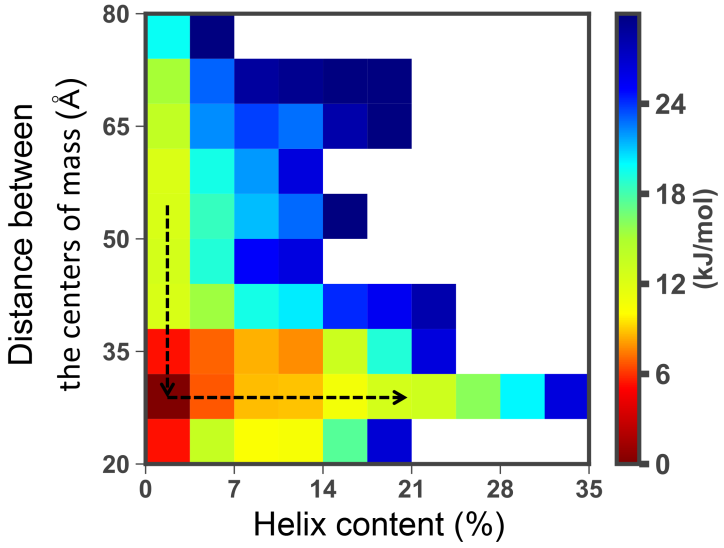

Figure S3. Two dimensional free energy landscape $F(d, c)$ as a function of helix content c, and the distance d between centers of mass of Aβ peptide and the GM1-glycan cluster. It was calculated by using all of the subsampled MD trajectories.

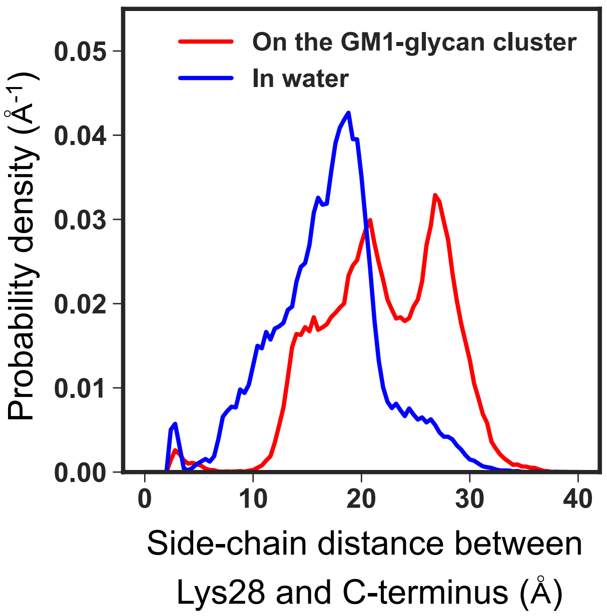

Figure S4. Side-chain distance between Lys28 and C-terminus in the cases of monomeric Aβ40 and Aβ40 on the GM1-glycan cluster. The side-chain distance was defined as a distance between the amino nitrogen atom of Lys28 and the center of mass of carboxylate oxygen atoms of C-terminus.

Supplementary Movie 1. A typical MD trajectory, in which Aβ40 forms a helix structure at C-terminus side (Red colored residue: Lys28, Blue colored residue: C-terminus, Green colored residue: Nue, Grey colored molecule: the GM1 glycan cluster except Neu).
